# Seeing a Blush on the Visible and Invisible Spectrum: A Functional Thermal Infrared Imaging Study

**DOI:** 10.3389/fnhum.2017.00525

**Published:** 2017-11-03

**Authors:** Stephanos Ioannou, Paul H. Morris, Marc Baker, Vasudevi Reddy, Vittorio Gallese

**Affiliations:** ^1^Department of Physiological Sciences, College of Medicine, Alfaisal University, Riyadh, Saudi Arabia; ^2^Department of Psychology, Centre for Situated Action and Communication, University of Portsmouth, Portsmouth, United Kingdom; ^3^Section of Human Physiology, Department of Neuroscience, Parma University, Parma, Italy; ^4^Institute of Philosophy, School of Advanced Study, University of London, London, United Kingdom

**Keywords:** thermal imaging, embodied cognition, blushing, compliment, physiology, shyness

## Abstract

So far blushing has been examined in the context of a negative rather than a positive reinforcement where visual displays of a blush were based on subjective measures. The current study used infrared imaging to measure thermal patterns of the face while with the use of a video camera quantified on the visible spectrum alterations in skin color related to a compliment. To elicit a blush a three-phase dialog was adopted ending or starting with a compliment on a female sample (*N* = 22). When the dialog ended with a compliment results showed a linear increase in temperature for the cheek, and forehead whereas for the peri-orbital region a linear decrease was observed. The compliment phase marked the highest temperature on the chin independent of whether or not the experiment started with a compliment contrary to other facial regions, which did not show a significant change when the experiment started with a compliment. Analyses on the visible spectrum showed that skin pigmentation was getting deep red in the compliment condition compared to the serious and social dialog conditions for both the forehead and the cheeks. No significant association was observed between temperature values and erythrocyte displays on the forehead and cheek. Heat is the physiological product of an arousing social scenario, however, preconceived notions about blushing propensity seem to drive erythrocyte displays and not necessarily conscious awareness of somatic sensations.

## Introduction

Emotions in humans have a strong behavioral and physiological bond with emotions in other primates. Yet, no animal has the same emotional display as that of a blush. Reddening of the upper chest, neck and face is an exhibition that appears, not only as originally believed in embarrassment, but also in pride, shame, guilt and shyness (Leary et al., 1992; Leary and Toner, 2012). At the core of these emotions is the interplay of the “self” and the “observer.” The individual exhibiting a blush is under a situation of severe self –awareness and self-concern in which an urge is being built for flight, however, the urge is not fulfilled due to potential social consequences (de Jong and Dijk, 2012). Blushing may be accompanied by an element of physical and emotional stress that seems to be important in social situations and interpersonal relationships, signaling trustworthiness as well as prosocial behavior (Leary and Meadows, 1991; Voncken and Bogels, 2009; Dijk et al., 2011). Nonetheless, blushing displays have received little attention regarding the physiological changes associated with the experience of a blush. The purpose of this paper is to explore physiological changes as a result of a compliment and to what extent temperature relates to visible alteration of skin tone.

Blushing seems to be driven by the social context in which an individual is engaged. This though is just one side of a double-edged sword as cognitive models of ourselves as well as personality characteristics seem to drive the intensity of the phenomenon. Drummond et al. (2003) documented that when individuals received a verbal feedback on their blush an increase in blood flow was observed further enhancing their social discomfort. Nevertheless this was evident only in individuals with increased blushing propensity according to their self-ratings. Results of the above-mentioned study suggest that preconceived notions formed by previous learning experience overshadow the feedback received by the physical sensations. This finds further support by the cognitive behavioral model of social anxiety (Clark and Wells, 1995). Individuals that are pre-occupied with their self-image attend first to the cognitive model of themselves and focus on their autonomic arousal (e.g., blood pressure, heart rate and temperature) further enhancing their blush and anxiety by believing that their self-impressions are also shared by others (Bögels and Mansell, 2004; Schultz and Heimberg, 2008). Miller (1996) argues that embarrassment or shyness stems from an individual’s belief about their “ideal self-image.” Actions of the individual out of the expected norm put the self-made “ideal image” into jeopardy creating a bad match for their social circle. This concept of how one appears in the eyes of others serves a much more general purpose and particularly the harmonious integration of the self into a social group (Baumeister and Leary, 1995). Individuals that do not function in response to “appropriate” social cues are socially ostracized and regarded as “strange” since they do not function within social norms (Campos, 1995; Fischer and Tangney, 1995).

Series of behavioral signs are exhibited during blushing. In the case of shyness these bodily displays function, at least for a brief period of time, to avoid communicative contact and prevent leaking of further affective cues to the observer (Izard and Hyson, 1986). One of the strongest behavioral indicators of shyness is the combination of gaze or head aversion together with a smile (Young and Decarie, 1977; Izard and Hyson, 1986; Lewis et al., 1989). The head or gaze usually moves downward (Lewis, 1995) and the smile has been characterized as closed-mouthed, with the lips being moderately pulled up and out, with no eyelid contraction (Reddy, 2000). A straightforward smile with eye contact may be too intimate in certain situations, especially on occasions where the audience is unfamiliar (Argyle and Dean, 1965). Moreover associated with shyness are behaviors such as low tone of voice (Buss, 1980) and an attempt to hide the mouth (Eibl-Eibesfeldt, 1989). However, there is very little physiological evidence associated with the physiological driving forces that lead to a blush (Kreibig, 2010).

Evidence on the physiology of facial blushing comes from patients with unilateral lesions on the sympathetic pathway of the face, which were subjected to a negative shyness scenario. These patients with damage on the 1st, 2nd, and 3rd cranial nerve of the brain stem exhibits a range of symptoms such as asymmetric facial flushing and sweating. To examine the implication of these neurons in affective physiology Drummond and Lance (1987) asked participants to sing children’s songs while their blood flow, capillary pulsation and facial perspiration was monitored. Although overall facial coloration (blushing) was observed in some patients (but not all), almost all participants had increased blood flow and sweating after the experimental condition was introduced. Unilaterally the lesion-affected region had significantly less physiological changes (of emotional sweating, vasodilatation and increased pulsation) showing that there is a positive association between cervical sympathetic pathways and facial flushing. Drummond (1997) in another attempt to explore the biological elements that lead to a blush observed that blocking of alpha or beta adrenoceptors was not enough to abolish vasodilation and had only a minor effect on a blush. These results lead him to conclude that localized vascular dilation may be associated with the release of endothelium relaxing factor (e.g., nitric oxide). Self-reports associate blushing with embarrassment and anger whereas facial pallor has been linked to fear (Drummond, 1997).

Blushing is an optical phenomenon and it stays within the “blush distribution” which includes the face, ears, neck and the upper chest (Wilkin and Richmond, 1988; Leary et al., 1992). Situated on the face this redness becomes evident when erythrocytes (hemoglobin) subcutaneously increases in quantity. Visible signs of a blush become evident approximately 15–20 s after the onset of the embarrassing incident (Shearn et al., 1990). The biological mechanisms driving this phenomenon are complex and no single physiological factor accounts for blushing (Leary et al., 1992; Hofmann et al., 2006). The physiology of blushing is poorly understood, however, studies so far point to three leading causes; vagal withdrawal that leads to heart acceleration (Hofmann et al., 2006); sympathetically mediated vasodilatation or vasoconstriction of specific subcutaneous blood vessels of the face (Drummond and Lance, 1987); and finally the presence of beta-adrenergic receptors selectively affecting vasodilation of specific facial vasculature (Mellander et al., 1982). Unlike any other body region, the face has more capillary loops and greater capacitance in subcutaneous blood vessels enabling greater blood flow (Wilkin and Richmond, 1988). Cheeks reveal such changes very clearly as the relevant vessels are much wider and closer to the surface of the skin without any fluid obscuring them (Ryan, 1973). From a psychophysiological perspective flushing or blushing increases the release of internal body heat by drawing blood to the surface of the skin (Rowell, 1977). But despite the possible homeostatic benefits, from an experiential perspective it is often regarded as a negative sensation (Zajonc et al., 1989). These physical changes may heighten the intensity of the aversive aspects of the embarrassing situation, but when flushing subsides this may signal the end of the experience (Drummond, 1999). The focus of this line of research has almost exclusively focused on blushing as a negative experience.

Methodological roadblocks restrict the study of self-conscious emotions (Lewis et al., 1989). Stimuli and films are less effective in reproducing a self-conscious emotional experience and it is difficult to imagine experimental protocols that are within ethical boundaries (Tracy and Robins, 2004). Putting the self in a “tight” social position seems to be the best method to arouse emotions such as embarrassment that are accompanied by a blush. Drummond (1999) asked female participants to perform a difficult task that was designed to allow only a 50% success rate regardless of what they did whilst being provoked by a confederate. Forehead pulse amplitude increased substantially after harassment whereas finger blood flow decreased indicating a physiological dissociation between distal body regions and facial vasculature. Thermal changes on the face are normally accompanied by color changes in the skin and there are associated changes in blood flow and vascular constriction or dilation. Nevertheless very few studies have investigated temperature changes during blushing and those that have did not produce consistent significant results (Mulkens et al., 1997, 1999; Edelmann and Baker, 2002; Drummond and Mirco, 2004). However, by inducing social pressure through social tasks, such as giving a speech in front of an audience, Voncken and Bogels (2009) observed temperature changes on the cheeks of participants. There was a linear increase in skin temperature during the embarrassment task. Moreover, participants with high levels of social anxiety had greater increases in skin temperature. The authors concluded that “self-focused attention on one’s blush may maintain and exacerbate blushing” (p. 93). Although not robust, similar effects have been obtained by Drummond and Mirco (2004). Subcutaneous blood flow as well as temperature was exaggerated on the right side of the face when individuals were being stared by the experimenter under an embarrassment-arousing experiment.

Thermal Imaging constitutes a novel approach in the arena of psychophysiology, where infrared light emitted naturally by the surface of the skin can be used as a measure of physiological changes that are a function of changes in affect. Functional infrared thermal imaging (fITI) bases the majority of observation on the thermal print of the face. The rich underlying vasculature makes the face an ideal candidate for monitoring peripheral physiology as autonomic changes in heart rate, muscular adaptation and epinephrine release in the blood stream are revealed in specific facial regions with unique temperature patterns (Ioannou et al., 2014a). Negative emotions such as fear (Nakayama et al., 2005; Ioannou et al., 2015; Kano et al., 2015) and guilt (Ioannou et al., 2013) have been associated with a decrease in nose temperature, a phenomenon associated with subcutaneous vascular constriction, evident on the extremities of the body and controlled by sympathetic efferent fibers (nose, ears, fingers, paws, and tail). On the contrary, social interaction (Ioannou et al., 2014b) and sexual arousal (Hahn et al., 2012) have been associated with a rather holistic temperature increase probably signaling the felt emotion. Moreover, on occasions of distress (such as mental workload or startle) perspiration pores increase in count (Pavlidis et al., 2012) leading to a temperature decrease on the upper orbicularis oris region (“Mustache”), whereas the peri-orbital region of the ocular cavity shows the opposite pattern, an increase in temperature (Pavlidis et al., 2002). Authors suggest that the local musculature that surrounds the eyes is responsible for the temperature increase of the peri-orbital region facilitating rapid saccades in case of a perceived threat. In fact Levine et al. (2001) observed a concomitant redistribution of thermal heat patterns in response to fear where the peri-orbital region showed an increase in temperature and the cheeks a decrease. The maxillary area (or the upper lip), although not among the regions of increased affective sweating (such as the axillae, palms, and soles of the feet), shows a positive correlation with the sweating of the fingers in response to startles. Thus inferences can be made about emotional arousal by observing the thermal signature of the upper lip (Shastri et al., 2009, 2012).

Like other physiological techniques thermal imaging has both advantages and disadvantages. Many conventional physiological measurement techniques pose restrictions to participants’ movements (Nakayama et al., 2005; Kuraoka and Nakamura, 2011) or require implantation of radio telemetric probes for autonomic monitoring (Vianna and Carrive, 2005). Most other techniques require some direct contact with the body restricting the types of experiments that can be conducted. Moreover, the use of temperature sensors on the skin is not an option as they get detached through contact, cover a small surface area and pressure on the skin can induce changes to regional blood flow affecting physiological recordings (Nakayama et al., 2005). fITI is a highly sensitive and versatile technique that converts infrared light into temperature allowing wireless monitoring of the participant (Ring and Ammer, 2012). The simplicity of using fITI for physiological monitoring makes it particularly useful in experimental designs that resemble real life situations in which liberty of movement is essential. For example self-conscious emotions are challenging to study in isolation (e.g., using films or photographs to elicit the emotion) as it is hard to substitute the social component with a stimulus presentation. Furthermore, established physiological techniques requiring body contact constantly remind the participant that is being monitored. The main physiological disadvantage of harnessing heat patterns on the surface of the skin is that unlike heart rate, temperature changes occur slowly and take approximately 10 s for a significant change to be observed (Kuraoka and Nakamura, 2011). Return to baseline values is even more troublesome as recovery time takes much longer than arousal and highly dependents on the region of interest (Vianna and Carrive, 2005). Moreover regarding the technical aspects of fITI unless the individual is relatively static so that a tracking algorithm can be applied manual extraction of data takes very long for moving subjects.

So far physiological signs of embarrassment have been identified using emotionally negative experimental paradigms while blushing propensity was assessed subjectively either by the participants experiencing the emotion or by the individuals observing the participant blush. The current study aims to examine blushing by using ecologically valid methods built into the experimental procedure. Thus thermal infrared imaging was used while employing a positive elicitor of shyness, a compliment given from a male to a female participant. By characterizing the thermal autonomic prints of blushing in adults, questions about theories on the physiology of a blush can be further understood and in extent examine how autonomic function affects temperature on the regions of the face. Moreover, although it is widely recognized that blushing has a thermal source we would like to examine to what extent are individuals aware of the physiological changes that occur on their face. Another novelty of the current study, in addition to observing thermal variations is that instead of basing blushing on subjective observations we quantified erythrocytes present on the surface of the skin using visible spectrum analyses. Furthermore we examined if blushing and thermal variations are related to each other.

## Materials and Methods

### Ethics Statement

The Research Ethics Committee of the Faculty of Science of the University of Portsmouth gave approval for the study. Experimental procedures were in line with the declaration of Helsinki and the Code of Human Research Ethics of the British Psychological Society.

### Participants

Twenty-two female participants aged between 19 and 23 years old (*M* = 19.86, *SD* = 1.73) were recruited for the study from a variety of cultural backgrounds. Participants originated from Scandinavia, South Eastern and Western Europe, the United States of America, Africa, Australia as well as South America. The major exclusion criterion was peripheral neuropathy. To improve the reliability of physiological observations, consumption of vasoactive substances such as nicotine, caffeine as well as alcohol was forbidden for at least 3 h prior to participation. The female participants were recruited through personal contacts and the University of Portsmouth recruitment database.

### Design

A 3 × 2 mixed factorial design was employed. The within subjects factor was type of social conversation (serious vs. social vs. compliment). The independent groups factor was the order in which the conversations took place (serious then social then compliment vs. compliment then social then serious). The dependent variable examined was skin temperature on six sites on the face (forehead, peri-orbital, nose tip, upper lip, cheeks, and chin).

### Procedure

Upon arrival individuals were asked to wait for 10 min in an acclimatization room until they were called in to participate. During that time they were briefed about the experimental protocol and were told that they were going to engage in a friendly dialog with the researcher while wirelessly being monitored with thermal imaging. Prior to entering the experimental room they completed an informed consent form as well as the BIS/BAS questionnaire (Carver and White, 1994). All female participants were requested not to wear makeup during the experimental procedure. Prior to the experimental procedure a circular ROI was placed on the nose and temperature was monitored for approximately 5 min during the 10 min acclimatization period. The nose was selected for establishing the pseudo-baseline as it is widely studied and most reactive to affective stimuli (Nakayama et al., 2005; Ioannou et al., 2014a). Once temperature did not fluctuate by more than ±0.1°C for a period of 60 s then experimental recordings started taking place. Baseline measures were taken by the infrared technician in the absence of the experimenter. The dialog with the experimenter was divided into three conditions (each flowing into the next), ‘serious,’ ‘social,’ and ‘compliment,’ with the order of presentation varied. Both the serious and the social conversation conditions were included in order to make the participant feel more comfortable with the experimenter as well as introduce a more natural social scenario for the main experimental variable which was the compliment. In Order 1 the ‘serious was followed by the ‘social’ and then the ‘compliment.’ In Order 2, the ‘compliment’ was followed by the ‘social’ and then the ‘serious.’ The ‘serious’ condition involved the following questions: “Why have you chosen the University of Portsmouth for your studies?”; “In which year are you currently in and what are you studying?”; “What modules have you attended this semester?”. The ‘social’ condition involved the following questions: “What do you enjoy doing on your free time?”; “What is your favorite color?”; “What was the best summer holiday that you had so far?. The ‘compliment’ condition involved the experimenter praising an aspect of the participant’s appearance, e.g., “I just noticed that the shape of your eyes is beautiful.” In all conditions the experimenter maintained attention toward the participant. Each condition took approximately 1 min and the whole experiment lasted 3 min allowing plenty of time for thermal changes to occur. It is important to note that after the compliment the experimenter averted his gaze from the participant and allowed at least 40 s after initiating the next dialog. During this short pause the experimenter pretended to be taking notes and this was performed in order not to physiologically contaminate the interaction with the participant. Once the experiment finished the participants completed a series of questions about the effectiveness of the experiment and then they were de-briefed about the aims and goals of the study.

### Materials and Data Acquisition

To record subcutaneous temperature variability a digital Guide Infrared TP8 camera (ThermoPro^®^) was used with an uncooled FPA microbolometer (384 pixels × 288 pixels). TP8 provides temperature sensitivity 0.08°Ñ at 30°Ñ and accuracy of ±1°C. Sampling rate was set at 1 frame per second (1 Hz). This was performed in order to generate enough frames that would balance out any potential movement artifacts by the participant. All recording took place approximately 50 cm away from the participants’ face and the camera was automatically calibrated and manually fixated to ensure a clean focal image. The experiment was always conducted between 3 and 5 p.m. and it was ensured that the participants were sat away from any sources of heat or direct wind currents. Prior to any recordings participants were left for approximately 10–15 min in the experimental room to acclimatize to the indoor temperature. In addition to temperature data, behavioral recordings were made by two radio-controlled video cameras (640 pixels × 480 pixels) with a frame rate of 50 Hz. The two video signals were combined using a Pinnacle system providing a two-split movie.

### Questionnaires

Skeptics in the topic of psychopathy postulate that primary psychopaths have weak behavioral inhibition traits (such feeling worried about making mistakes) and low fear responsiveness (Lykken, 1995). In secondary psychopathy these traits are rather normalized in contrary to high levels of impulsivity and sensation seeking despite future regrets (Hughes et al., 2012). Behavioral exhibitions of shyness relate to enhanced pro-social skills (Dijk et al., 2011), social anxiety (Dijk et al., 2009) and in extent worry about the evaluation of others (de Jong and Dijk, 2013). Thus it is important to exclude personality characteristics such as psychopathic traits that may influence the variable of interest. To control for any personality variables that might have been influencing autonomic arousal and response in complex social situations the BIS/BAS scale by Carver and White (1994) was administered, For the current study a two-factor model was followed where BIS was divided into two subscales BIS-anxiety, (four items) related to conflict and negative criticism as well as the fight/flight/freeze system (FFFS-fear) that relates to fear responsiveness and punishment (Heym et al., 2008). Three subscales make up the BAS scale (a) Drive for achieving goals (DR-4 items), (b) Fun-Seeking or Sensation Seeking (FS-4 items) and (c) Reward Responsiveness (RR-5 items). Frequency analyses showed that 79.5% of participants scored above average for BIS and 52% above average for BAS thus according to the literature the majority of the population sample had no primary or secondary psychopathy traits (Hughes et al., 2012). For the current study Cronbach’s alpha value were for *BIS-Anxiety* 0.53, for *FFFS-Fear* 0.52, *BAS-Drive* 0.67, *BAS-Fun Seeking* 0.32, and *BAS-Reward Responsiveness* 0.69. Despite the small alpha values this psychometric scale is one of the most widely established personality measures and has good discriminant and convergent validity (Campbell-Sills et al., 2004). Three further questions were given to the participants regarding their personal experience during the experimental session. Ratings were taken on a five point Likert-Scale. For question (A) (When you were complimented did you feel shy?) answers ranged from 1 = not shy at all to 5 = very shy, for question (B) (When you were complimented did you feel uncomfortable?) answers ranged from 1 = not uncomfortable to 5 = very uncomfortable, and for question (C) (When you were complimented did you feel that your face was getting warmer?) answers ranged from 1 = not warm at all to 5 = very warm. Collected scores were averaged to examine if the experimental manipulation was successful and all scores were above the median value. In extent a paired sample *t*-test was conducted to examine if there was a significant difference between order 1 and order 2. Lastly, diagrams of faces were given to the participants and they were asked to mark with an asterisk where they had felt elevations in temperature after the compliment.

### Experimental Manipulation

Subjective scores in emotional and physical domains of the experiment were analyzed in order to observe the success rate of the experimental manipulation as well as examine whether or not the order in which the dialog took place affected embarrassment scores. Overall subjective levels of shyness were above average with 31.8% of participants scoring 5 (very shy), 31.8% of participants scoring 4, 27.3% of participants scoring 3, and 9.1% of participants scoring 2. Subjective ratings on levels on unease after a compliment had equivalent rates of success as 9.1% felt very uncomfortable (5), 50% scored above average (4), 27.3% experienced medium levels of unease (3) and 13.6% felt slightly uncomfortable (2). The final question regarding the warmth of the face after the compliment had 72.7% of values above average of which 31.8% scored very warm and 40.9% quite warm. The rest of the scores were 4.5% for not warm at all (1) and 22.7% for somewhat warm (2).

### Thermal Data Analyses

Prior to any extraction of temperature data both the behavioral and thermal recordings were synchronized in order to represent the same point in time. The experiment was separated into experimental segments according to the order in which it was conducted and thermal data was extracted from six regions of interest: the chin, cheek, nose, maxillary, forehead as well as peri-orbital region. Thermal data collection was performed using Launch GuideIR Analyser software by Wuhan Infrared Technology^1^. A multitude of shapes was used to extract the average temperature from the region of interest such as rectangular for the maxillary area and the forehead, circular for the cheek, nose and the peri-orbital region and oval shapes for the chin (Ioannou et al., 2014a). It was ensured that there was no variability across frames in the shape of the ROI and the placement of the region of interest. Any variability in these parameters disturbs the average value of the recorded temperature. This was ensured using anatomical landmarks as well as constantly monitoring the degree of change from one frame to the next (not larger than >0.2°C) (Ioannou et al., 2014a). Temperature was recorded every 5 s ensuring that micro-movements by the participant did not induce any large fluctuation in skin temperature > 0.2°C. One of the advantages of manual data extraction is the fact that it allows you to control for movement artifacts by looking at large jumps in temperature (<0.5) within the 5 s time frame. If these artifacts are induced usually they result from a change in the angle of facial recordings. In the occasion that an artifact was recognized then the next available frame (6–15s) was used or the previous one (4 s). In the occasion that an artifact entered the main data set for a particular individual the outlier was eliminated by running a histogram to check for an uneven distribution. Then the problematic frame was reanalyzed. Stem and leaf plots were run on the whole data per individual to ensure that no outliers have entered the analyses. On average 43 frames were extracted for each participant, approximately 14 for each condition. These 14 frames were first averaged for each individual prior to conducting any group analyses. To perform the analyses the Statistical Package for the Social Sciences, version 17 (SPSS, Chicago, IL, United States) was used. To ensure that data was suitable for parametric analyses a reliability test was conducted. The second rater placed regions of interest on pre-selected frames by the first rater and performed the analyses again of five individuals (43× frames). Then a 2 × 5 ANOVA was conducted in order to examine if there were any differences between the two raters. No significant difference (*p* < 0.05) was observed between the scores of rater 1 and rater 2. For rater 1 the average degree of change for the nose was *M* = 0.33 *SD* = 0.43, for the maxillary *M* = 0.06, *SD* = 0.38, for the peri-orbital *M* = -0.39, *SD* = 0.36, the chin *M* = -0.14, *SD* = 0.34, the forehead *M* = -0.02, *SD* = 0.24 and the cheek *M* = -0.23, *SD* = 0.13. For rater 2 the nose was *M* = 0.33 *SD* = 0.43, for the maxillary *M* = 0.06, *SD* = 0.38, for the peri-orbital *M* = -0.39, *SD* = 0.35, the chin *M* = -0.14, *SD* = 0.33, the forehead *M* = -0.02, *SD* = 0.24 and the cheek *M* = -0.22, *SD* = 0.12. Moreover a high degree of reliability was found between Rater 1 and Rater 2 for all region of interest. The average measure of intraclass correlation coefficient for all regions of interest was 1, *p* < 0.001. To examine the effect of the compliment on facial temperature a 3 × 2 mixed MANOVA was conducted where the repeated measures factor was condition (serious, social, and compliment) and the independent groups factor was order of presentation (serious, social, compliment vs. compliment, social, serious); the dependent variables were the six ROIs on the face (forehead, nose, maxillary, periorbital, cheek, and chin). Non-Parametric Spearman correlations were performed for the six regions of interest (chin, forehead, cheek, maxillary, periorbital, and nose) based on the experimental order. Moreover in order to examine which region of the face marked the highest temperature during the compliment phase a 6 × 2 mixed MANOVA was conducted using the six different ROIs as the repeated measures factor and the two orders as the independent group factor (serious, social, compliment vs. compliment, social, serious).

### Visible Spectrum Analyses

To detect visible color changes on the face we used ImageJ analyzer http://imagej.nih.gov/ij/index.html to extract pixel values across different conditions. Only the red channel was taken into account. Prior to performing any type of analyses we collected the responses given by the participants on the facial diagram and followed the area, which they indicated to be getting warmer after the compliment phase. Out of the 22 participants only one of them reported no temperature change on any region of the face. The rest, 21 participants, indicated predominantly the cheeks. Along with the cheeks one individual indicated the ears and another participant the forehead. The cheek was established as an area of common interest for all participants in addition to the forehead in which the majority of the experimental literature has repeatedly indicated this region to be implicated in embarrassment and shyness (Mulkens et al., 1997, 1999; Drummond, 1999; Edelmann and Baker, 2002; Drummond and Mirco, 2004). Behavioral videos were divided into experimental segments. In total, three frames were extracted for each individual based on the experimental condition (Serious, Social, and Compliment) and were selected 30 s after the initiation of the experimental condition. All images were saved as a portable networks graphics (.png) and then opened in ImageJ software for further analyses. Circular ROIs were drawn on the images with identical shape and radius. As for the infrared images visible spectrum analyses was being done using anatomical landmarks. Pixel values were measured on a red light color bar (between 0 and 255) and recordings were taken on the mean intensity of the pixels within the circle’s radius as well as the standard deviation. To examine if there was a significant difference in the mean scores of facial coloration 3 × 2 mixed MANOVA was conducted for both the cheeks and the forehead. The repeated measures factor was Condition (serious vs. social vs. compliment) and the independent groups factor was Order (serious then social then compliment vs. compliment then social then serious). Once this was established, a Spearman correlation was performed to determine if there was an association between temperature values and color of the cheeks as well as the forehead. In extent a Non-parametric Spearman correlation was performed between the redness values of the forehead the cheek. To examine whether analyses on the visible light spectrum was the result of natural sunlight a spectrum analyses was conducted within the experimental room. The ROI, which was placed on the same side in the room as the ROI of the face showed no significant light alteration throughout conditions (*p* > 0.05).

## Results

### Temperature Analyses

There was no significant multivariate main effect of order. There was an overall significant multivariate effect of condition, λ = 0.90, *F*(12,9) = 6.97, *p* = 0.003, $\eta_{p}^{2}$ = 0.90, with a large effect size. However, this was qualified by a significant multivariate condition × order interaction, λ = 0.87, *F*(12,9) = 5.81, *p* = 0.006, $\eta_{p}^{2}$ = 0.72 with large effect size. To confirm that our results had significant power to detect significant interaction effects between condition and order a *post hoc* power analyses was performed using the G^∗^Power software (Faul et al., 2007). Having a sample size of 22 and an eta squared of 0.72 the study had enough power to detect multivariate condition interactions at a more than an adequate power level (^∗^0.80) with a critical *F*(12,9) = 3.52. Follow up univariate tests revealed significant main effects of condition for forehead, periorbital, cheek and chin, and significant condition × order interactions for forehead, periorbital and cheek (see **Table 1**).

**Table 1:** Results of univariate ANOVAs on individual ROIs.

ROI	Source	*F*(df)	*P*	$\eta_{p}^{2}$	90% CI $\eta_{p}^{2}$
Forehead	Condition	**20.25 (2,40)**	**<0.001**	**0.50**	**0.29–0.61**
	Order	0.004 (1,20)	0.95	0.00	
	Condition × Order	**5.67 (2,40)**	**0.007**	**0.22**	**0.04–0.36**
Nose	Condition	0.39 (1.25,25.05)	0.58	0.02	
	Order	0.35 (1,20)	0.56	0.02	
	Condition × Order	3.47 (2,40)	0.07	0.15	
Maxillary	Condition	1.77 (2,40)	0.18	0.08	
	Order	0.26 (1,20)	0.62	0.01	
	Condition × Order	0.150 (2,40)	0.86	0.01	
Periorbital	Condition	**4.07 (2,40)**	**0.02**	**0.17**	**0.01–0.31**
	Order	1.88 (1,20)	0.29	0.06	
	Condition × Order	**4.67 (2,40)**	**0.015**	**0.19**	**0.02–0.33**
Cheek	Condition	**5.71 (1.50,30.05)**	**0.01**	**0.22**	**0.03–0.39**
	Order	2.02 (1,20)	0.17	0.09	
	Condition × Order	**11.83 (1.50,30.05)**	**<0.001**	**0.37**	**0.13–0.52**
Chin	Condition	**4.58 (2,40)**	**0.016**	**0.17**	**0.01–0.30**
	Order	1.26 (1,20)	0.27	0.06	
	Condition × Order	2.36 (2,40)	0.12	0.09	

*Significant effects are indicated in Bold font.*

**FIGURE 1:**
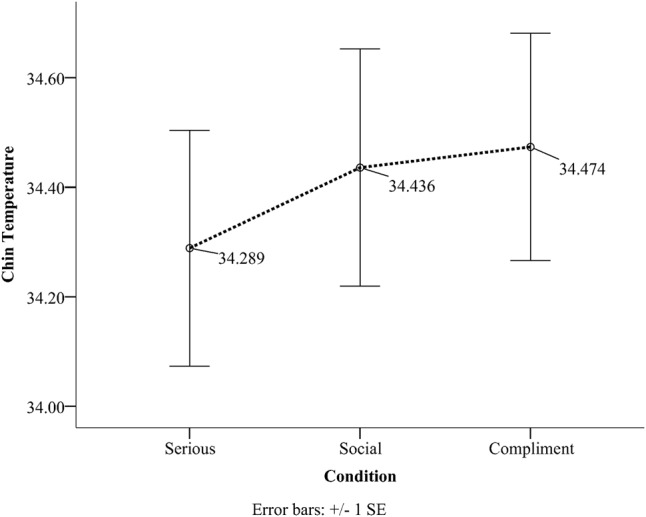
Chin temperature as a function of condition.

We only interpret the main effect for chin as there was no interaction for this ROI. No significant condition × order interactions was observed for the chin. For the chin, the serious condition (*M* = 34.29, *SD* = 1.01) had the lowest temperature and the compliment condition (*M* = 34.47, *SD* = 0.97) had the highest temperature compared to the social condition (*M* = 34.43, *SD* = 1.02) (see **Table 1** and **Figure 1**). The difference between the serious condition and the compliment condition was significant (*p* = 0.02, *M*^diff^ = 0.18).

**FIGURE 2:**
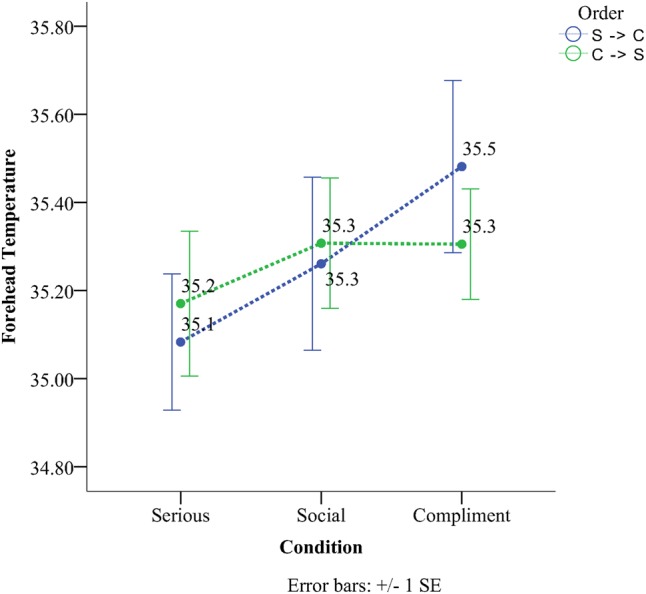
Temperature of forehead as a function of condition and order.

There was a significant condition × order interaction for the forehead. As can be seen from **Figure 2**. This was due to the fact that in Order 1, (SSC, i.e., Serious, Social, and Compliment) there was a significant increase in temperature across conditions; all comparisons between conditions were significant (*p* < 0.05), (see **Figure 2**). The serious condition (*M* = 35.08, *SD* = 0.51) had the lowest temperature and significantly differed (*p* = 0.02, *M*^diff^ = 0.18) from the social condition (*M* = 35.26, *SD* = 0.65) as well as the compliment (*M* = 35.48, *SD* = 0.65) condition (*p* < 0.01, *M*^diff^ = 0.39). The compliment condition had the highest temperature and significantly differed from the social condition (*p* = 0.002, *M*^diff^ = 0.22). In contrast there were no significant differences in temperature across conditions in the order CSS.

**FIGURE 3:**
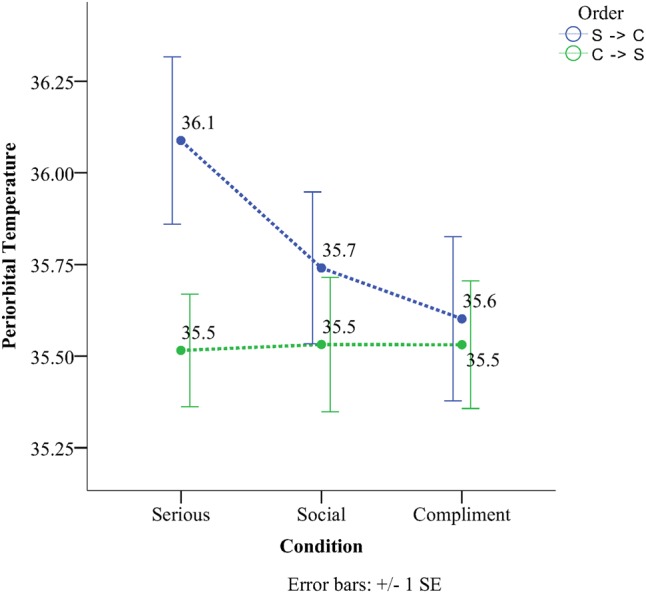
Temperature as a function of condition and order in the periorbital region.

There was a significant order × condition interaction for the periorbital region. The interaction for the peri-orbital region (see **Figure 3**) is explained by the fact that in the order SSC the serious condition (*M* = 36.10, *SD* = 0.76) was significantly higher than both the social condition (*M* = 35.74, *SD* = 0.69) (*p* = 0.02, *M*^diff^ = 0.35) and the compliment condition (*M* = 35.6, *SD* = 0.74) (*p* = 0.02, *M*^diff^ = 0.49). In contrast there were no differences between conditions in the order CSS.

**FIGURE 4:**
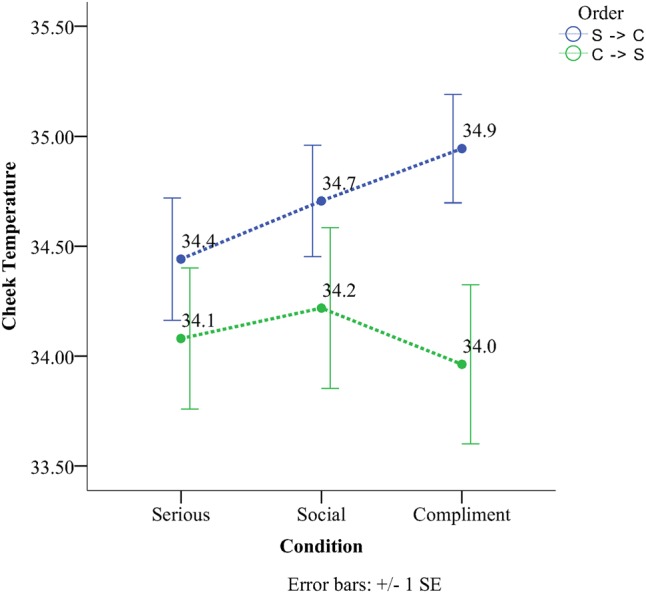
Temperature as a function of condition and order in the cheek region.

There was a significant Condition × Order interaction for the cheek scores (see **Figure 4**). In the SSC, all conditions were significantly different from one another (all *p* < 0.05) with the serious condition having the lowest temperature (*M* = 34.44, *SD* = 0.92), followed by the social condition (*M* = 34.44, *SD* = 0.92) and lastly the compliment condition (*M* = 34.94, *SD* = 0.82). The serious condition significantly differed from the social (*M*^diff^ = 0.26, *p* < 0.01) as well as the compliment condition (*M*^diff^ = 0.50, *p* < 0.01). The compliment condition was higher by (*M*^diff^ = 0.24, *p* = 0.03) than the social phase condition (see **Figure 4**). In the order CSS there were no differences between conditions.

Non-Parametric Spearman correlations according to order for the six regions of interest yielded significant results for the majority of facial regions. All reported values in **Table 2** have been Benjamin-Hochberg adjusted.

**Table 2:** Results of Spearman’s correlations based on the two orders SSC and CSS on the six ROIs.

	Nose	Maxillary	Periorbital	Cheek	Chin
**SSC**					
Forehead	0.306	0.453^∗^	0.332	0.400^∗^	0.718^∗∗^
Nose		0.518^∗∗^	0.403^∗^	0.305	0.586^∗∗^
Maxillary			0.797^∗∗^	0.535^∗∗^	0.749^∗∗^
Periorbital				0.166	0.562^∗∗^
Cheek					0.435^∗^
**CSS**					
Forehead	0.142	0.520^∗∗^	0.345^∗^	0.474^∗∗^	0.334
Nose		0.500^∗∗^	–0.116	0.145	0.515^∗∗^
Maxillary			0.196	0.436^∗^	0.504^∗∗^
Periorbital				0.293	0.577^∗∗^
Cheek					0.405^∗^

*^∗∗^Correlation is significant at the 0.01 level (2 tailed). Benjamin–Hochberg adjusted. ^∗^Correlation is significant at the 0.05 level (2 tailed). Benjamin–Hochberg adjusted.*

Analyses of variance for the six regions of interest showed no temperature difference between the SSC and CSS order *F*(1,20) = 0.005, *p* = 0.94, $\eta_{p}^{2}$ = 0.00. No significant region × order interaction across *λ* = 0.66, *F*(5,16) = 1.65, *p* = 0.203, $\eta_{p}^{2}$ = 0.34. There was, however, a significant main effect for the six regions of interest *λ* = 0.24, *F*(5,16) = 10.16, *p* = 0.001, $\eta_{p}^{2}$ = 0.76 (**Table 3**). The peri-orbital region had the highest temperature (*M* = 35.6, *SD* = 0.65) and the nose the lowest (*M* = 31.28, *SD* = 3.03). The forehead had the second highest temperature (*M* = 31.28, *SD* = 3.03) followed by the maxillary (*M* = 34.82, *SD* = 0.81), the chin (*M* = 34.47, *SD* = 0.97) and the cheek (*M* = 34.45, *SD* = 1.12).

**Table 3:** Main effect analyses showing the differences in temperature for the six regions of interest.

	Nose	Maxillary	Periorbital	Cheek	Chin
Forehead	4.11^∗∗^	0.57^∗^	–0.17	0.94^∗∗^	0.92^∗∗^
Nose		–3.54^∗∗^	–4.28^∗∗^	–3.17^∗∗^	–3.19^∗∗^
Maxillary			–0.74^∗^	0.37	0.35
Periorbital				–1.11^∗^	1.09^∗∗^
Cheek					–0.02

*^∗∗^Mean difference is significant at the 0.01 level (2 tailed). Sidak adjusted. ^∗^Mean difference is significant at the 0.05 level (2 tailed). Sidak adjusted.*

### Subjective Question Analyses

In order to examine if there was a significant difference between the ratings of the participants who were exposed to Order 1 (serious, social, and compliment) compared to Order 2 (compliment, social, and serious) a paired sample *t*-test was conducted. The first question regarding how shy participants felt during the experiment had no significant difference between Order 1 (*M* = 4.18, *SD* = 0.87) and Order 2 (*M* = 3.55, *SD* = 1.04), *t*(10) = 1.41, *p* = 0.19. The mean increase from Order 1 to Order 2 was 0.64 with a 95% confidence interval ranging from -0.37 to 1.65, η^2^ = 0.18. The second question regarding how unpleasant the compliment was yielded a significant difference between Order 1 (*M* = 4.00, *SD* = 0.63) and Order 2 (*M* = 3.10, *SD* = 0.83), *t*(10) = 2.65, *p* = 0.024. The mean increase was 0.9 ranging from 0.15 to 1.67, η^2^ = 0.41. No difference was observed between Order 1 (*M* = 4.10, *SD* = 1.22) and Order 2 (*M* = 3.81, *SD* = 0.63), *t*(10) = 0.64, *p* = 0.54 regarding facial warmth. The mean increase was 0.27 ranging from -0.68 to 1.23, $\eta_{p}^{2}$ = 0.04.

### Visible Spectrum Analyses

**FIGURE 5:**
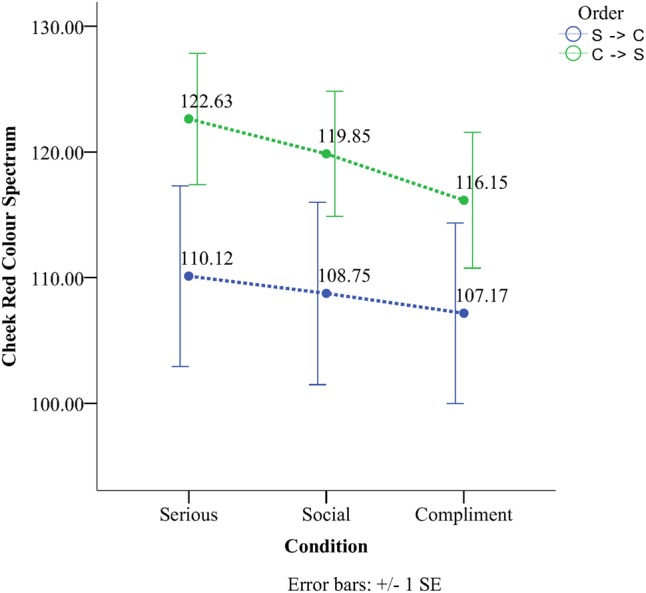
Red Color Spectrum as a function of condition and order.

For the cheeks there was a significant main effect of Condition λ = 0.32, *F*(2,40) = 25.38, *p* < 0.001, $\eta_{p}^{2}$ = 0.56, there was also a significant Condition × Order interaction, λ = 0.77, *F*(2,40) = 3.57, *p* = 0.037, $\eta_{p}^{2}$ = 0.15, but it was ordinal justifying analysis of the main effect of Condition. Main effects analyses for Condition revealed that all conditions were significantly different from all others (*p* < 0.01). The compliment had the darkest skin tones (*M* = 111.66, *SD* = 4.86), the social condition showed the next darkest skin tones (*M* = 114.29, *SD* = 20.93) and the lightest skin tones were present during the serious condition (*M* = 116.37, *SD* = 21.33). As can be seen from **Figure 5** the interaction was the result of a larger difference between conditions in the CSS order than the SSC order. Simple main effects analysis confirmed this interpretation. All of the differences between conditions were significant in the order CSS (*p* < 0.01), however, in the SSC order the only significant difference was between the serious and compliment condition (*p* = 0.033).

The forehead did not show a significant Condition × Order interaction, λ = 0.82, *F*(2,40) = 2.17, *p* = 0.12, $\eta_{p}^{2}$ = 0.17, but a main effect for condition was observed λ = 0.33, *F*(2,40) = 18.82, *p* = 0.001, $\eta_{p}^{2}$ = 0.66. Main effect analyses for Condition revealed that all conditions were significantly different from each other (*p* < 0.01). The compliment had the darkest skin tones (*M* = 106.7, *SD* = 19.10) followed by the social phase (*M* = 109.8, *SD* = 19.39) and the serious phase (*M* = 112.32, *SD* = 19.51).

The relationship between facial coloration and temperature was investigated. We correlated color and temperature for the cheek and for the forehead; we conducted separate analyses for the two orders (CSS; SSC) for each condition (serious, social, and compliment). This meant that each analysis was based on 11 participants. We found that 10 of the 12 correlations were negative ranging from -0.24 to -0.57. However, as none of the correlations alone were significant the results must of course be treated with caution. But given that the result is counterintuitive we thought it worth reporting as a basis for further study.

### Thermogram Description

**FIGURE 6:**
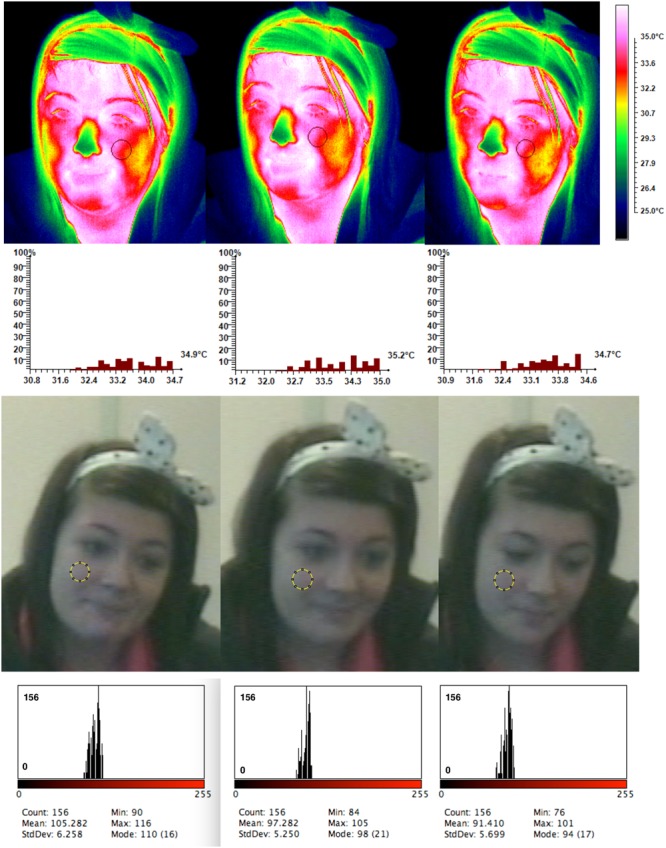
Temperature throughout conditions and cheek color change for the CSS order.

**Figure 6**: Images selected for the illustration of results are representative of the general tendency of the group sample. When the experiment started with a serious dialog a very prevalent qualitative change was observed on the cheeks. Whereas the majority of the area is covered with green shades during the serious dialog these shades move to overall yellow with apparent red patches on the social conversation. These red marks become even more apparent in the compliment phase. The chin shows a similar tendency in which green to yellow shades move to red on the social phase and even white on the compliment. In the current infrared image certain regions on the infrared spectrum do not show visible changes due to the range of temperature values selected for the specific palette. Lowering or increasing the infrared range would compromise the visible changes on specific facial region in favor of others. To avoid this illustration problem one needs to segment regions of interest to a specific temperature range. This can be achieved with FLIR Researcher IR compatible only with the use of FLIR cameras.

**FIGURE 7:**
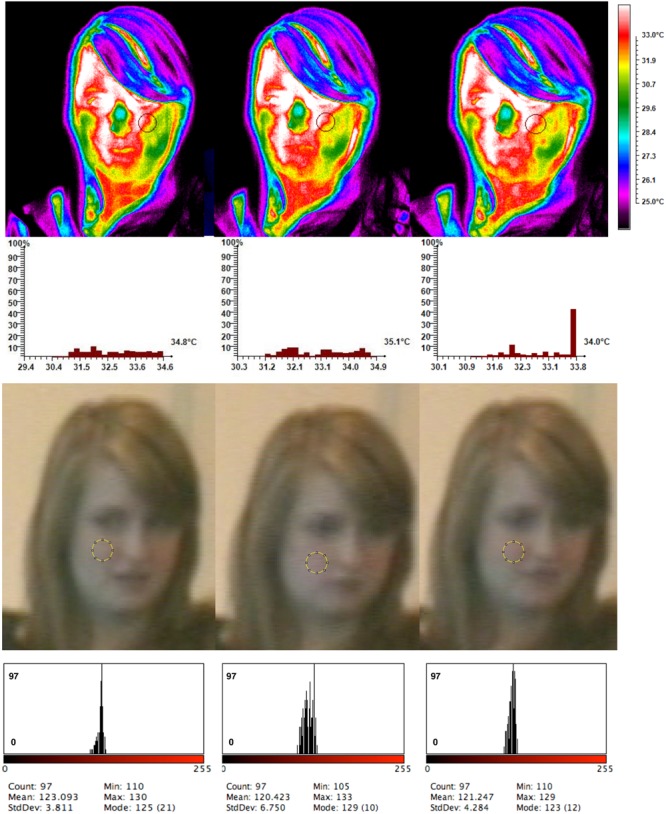
Temperature throughout conditions and cheek color change for the SSC order.

**Figure 7**: Moving to the next experimental order when the experiment started with a compliment an increase in shades of red were observed on the cheeks from the compliment phase to the social phase. More yellow shades seem to be dominant on the last phase of the CSS order on the cheeks. Very subtle color changes seem to occur on the forehead throughout phases but the last phase seems to have the least concentration of white shades.

## Discussion

### Summary

The current study examined the effect of compliments on subcutaneous facial temperature and investigated the relationship between facial temperature and facial coloration. Data analyses for the social, then serious and finally compliment order (SSC) revealed a significant linear temperature increase on the forehead, the cheek, and the chin compared to the periorbital region that showed a significant linear decrease. When the experiment started with a compliment and ended with a serious conversation (CSS) chin temperature still showed the same tendency (as the SSC order) with the serious condition having the lowest temperature and the compliment one the highest. The same result as the chin was not observed for the remainder of the ROIs concerning the CSS order. Temperature in the periorbital region remained rather stable throughout the different orders. Facial skin analyses showed that more red skin tones were present during the compliment condition and more fair ones during the serious condition for both the forehead and the cheeks. This effect persisted independently of order. Temperature on the other hand across the six different regions yielded a more selective set of results (**Table 3**). Thermal comparison between the six sites of interest during the compliment phase showed that the peri-orbital region had the highest temperature and the nose the lowest.

### Infrared Spectrum

Various scientists for the study of shyness and in extent blushing have employed different experimental paradigms. Experimentally it has been shown that social pressure in the context of public performance (Hofmann et al., 2006; Voncken and Bogels, 2009) as well as humiliation (Drummond, 1999) causes an increase in blood flow on the cheeks and forehead (Drummond, 1999; Voncken and Bogels, 2009), an increase in skin conductance (Drummond, 1999; Hofmann et al., 2006; Voncken and Bogels, 2009), as well as reduced respiratory sinus arrhythmia (Hofmann et al., 2006). Nevertheless there is a common meeting point in all. Blood flow increase directly links to subcutaneous temperature changes (Drummond and Mirco, 2004; Vianna and Carrive, 2005; Voncken and Bogels, 2009).

Throughout the experiment most of the regions of the face showed a linear increase in temperature when the experiment started from the serious condition and continued to the compliment phase. However, when the experiment started from the compliment and moved into the social condition temperature remained rather stable. Only for the cheeks the social phase had a higher temperature nevertheless this did not reach statistical significance. Voncken and Bogels (2009) observed the same phenomenon on the cheeks using thermistors moving from the social task to the recovery phase. This phenomenon, as has been previously documented, is caused by a delay in subcutaneous tissue not only to heat up but also to cool down (Vianna and Carrive, 2005). Ioannou et al. (2014b) have previously observed this phenomenon when conditions of high physiological arousal follow others of lower affective value and have adopted the name *“physiological spill-over effect.”* Clearly it is difficult to exclude the fact that presenting someone with a sincere compliment in a socially appropriate moment differs from a compliment on a particular personal feature during an interview with a senior faculty member; at the very least it is a far more ambiguous behavior/situation and could be easily interpreted as an inappropriate act of seducing a participant. Such interpretation might also help explain why in the order that started with the compliment condition there was no differential effect of condition because the inappropriate compliment might have provided an awkward context for the entire experimental procedure.

The chin on the other hand did not show any order effect and the rise in temperature was attributed to the effect of the condition. One of the reasons that this effect may have taken place could be the fact that the chin is the first facial region that receives direct input from the external carotid, thus it is likely to be more susceptible to direct alteration of cardiac output. Nevertheless we cannot exclude the fact that temperature increase may be partly due to gnathic muscle action. Based on the subjective ratings none of the participants mentioned the chin as an area that was getting hotter. This observation needs further exploration to be adequately addressed but it is more than likely that self-reports on somatic sensation at least in the area of shyness and blushing are more likely to be driven by social feedback rather than conscious awareness of physiological changes. Thus the somatic map of emotions created by Nummenmaa et al. (2013) needs to seriously recognize the limitation associated with subjective ratings.

The peri-orbital region was one of the few facial sites that showed a decrease in temperature. In the majority of thermal imaging studies increments in temperature on the forehead are associated with increments on the periorbital region (Levine et al., 2001; Puri et al., 2005; Ioannou et al., 2014b). Current subjective observations support the opposite effect in terms of temperature tendencies. Nevertheless exploratory correlation analyses did not yield a negative relationship between the forehead and the cheek during the compliment phase, not even when the other two phases of the experiment (serious and social) were taken into consideration. Looking deeper into the experimental literature it seems that microvasculature of the face is controlled separately for each region and according to environmental demands. This is not far from true based on the ipsilateral effects of blood flow as a result of gaze observed by Drummond and Mirco (2004). Levine et al. (2001) observed that when participants were startled, temperature on the cheeks dropped while temperature on the peri-orbital region rose. Here the opposite effect was observed. The peri-orbital region cooled down while the temperature on the cheeks rose. According to Leary et al. (1992) embarrassment appears quickly in the form of short blushes and then disappears. Such as the startle reflex the blush may also represent a rapid redistribution of blood to signal the felt emotion and then a quick recovery to baseline values.

Correlation analyses across the six regions of interest did not lead to a significant association uniformly across all facial sites. The reason for the above result is the fact that certain blood vessels are closer to the surface of the skin such as the dorsonasal vessels of the periorbital region and the supraorbital vessels of forehead compared to the infraorbital vessel of the cheeks. In fact the dorsonasal and supraorbital vessels are so visible that makes them ideal candidates for wireless polygraph testing (Tsiamyrtzis et al., 2006; Zhu et al., 2007). Another factor affecting the relationship across the different regions of interest is the difference in subcutaneous tissue compositions (Pavlidis et al., 2007). Certain regions may have more percentage of fat and muscle thus in addition with vessel positioning a difference in tissue warming would be the result. The uneven warming up period across the six facial regions is possibly the best explanation behind current scientific findings.

### Visible Spectrum

In many cultures it is accepted that blushing is an indication of emotional arousal. Whether blushing represents anger or embarrassment one cannot really tell without first interpreting the source of arousal (Schachter and Singer, 1962; Drummond, 1997). Here instead of using subjective observer rating measures (Voncken and Bogels, 2009) a color spectrum analysis was performed on the cheeks as well as the forehead. As the conversation between the experimenter and the participant became more personal, skin color moved to lower values of the red color spectrum. Independent of order, skin pigmentation reached more red values during the compliment phase and was fairer during the social conversation. Skin color and temperature showed a significant correlation during the CSS order only for the forehead. Three reasons might account for this phenomenon. Color analyses on the visible spectrum may be sensitive enough to represent physiological changes corresponding to color in real time values. Second, temperature development is sluggish so heat emission and color may be on this occasion unrelated as they have different onsets. Changes in color of the face may appear due to rapid dampening of sympathetic tone leading to a relaxation of arterioles and rapid blood influx to the skin surface signaling the felt emotion. During a blushing episode blood perfusion to the facial tissue may be isolated (at least in its majority) to the superficial layers of the skin deposited there by fine arterioles. This physiological mechanism may explain why in certain experimental paradigms there is a moderate association between blood perfusion and temperature (Mulkens et al., 1997, 1999; Edelmann and Baker, 2002; Drummond and Mirco, 2004). Third the fact that supraorbital vessels (as mentioned) are close to the surface of the skin in addition to the potentially awkward experimental order in which the compliment preceded the social discussion could have led to a stronger physiological response. Nevertheless this phenomenon needs further exploration to be confirmed as this is the first time that pixel analysis has been used in conjunction with thermal imaging. Blushing is possibly caused by a rapid dilation of subcutaneous vessels either in response to increased cardiac output and pressure or a drop in sympathetic vasoconstrictor tone. Heat conduction of the skin is sluggish. This effect will take place gradually and it will intensify by increases in cardiac output. One potential solution to overcome a problem as such would be to start measuring thermal changes of affective states from 15 s (Kistler et al., 1998) to 50 s (Nakayama et al., 2005) after stimulus manipulation in order to account for the thermal delay phenomenon.

### Subjective Ratings

The experience of a blush has been suggested to be a rather emotionally stressful experience despite serving a pro-social adaptive strategy (Leary and Meadows, 1991; Voncken and Bogels, 2009; Dijk et al., 2011). In agreement with the above observation are the subjective ratings received by the participants since they rated the compliment phase as making them feel not only shy but also uncomfortable. Previous studies have shown similar effects with self-beliefs about a challenging social scenario driving erythrocyte displays (Bögels and Mansell, 2004; Schultz and Heimberg, 2008).

### Physiological Reflection on Blushing

There is no consistent account in the experimental literature regarding the physiological driving forces that lead to a blush. Evidence suggests that vasodilation during blushing is not generalized across the body (Frijda, 1986; Drummond, 1999). Instead it is a rather selective process involving the face, ears, neck, and upper chest, supporting both communicative and remedial approaches (Leary et al., 1992). The classic blush appears rapidly on the face increasing the accumulation of blood volume subcutaneously through the facial vasculature, withholding more blood than any other region of the skin (Wilkin and Richmond, 1988). Patients with sympathetic lesions (e.g., suffering from Horner’s syndrome) have diminished blushing propensity at the site of the affected lesion. Vascular dilation of facial blood vessels has been proposed by Mellander et al. (1982) to be mediated by local beta-adrenergic influence that respond to factors or stimuli that other arterioles do not (as cited by Leary et al., 1992). These receptors seem to decline with age and are inversely related to blushing propensity (see for the opposite Drummond, 1997). Shyness and social anxiety have usually been examined in conjunction, but they have distinct psychophysiological functions (Hofmann et al., 2006). Unlike anxiety, what characterizes embarrassment is immobility rather than arousal. Shyness has been associated with an increase in heart rate and despite being lower than anxiety it is still higher than baseline values (Hofmann et al., 2006). By collectively taking into account the experimental literature in addition to our findings it is possible to argue that blood may be redirected locally from regions of less functional significance (such as the ocular cavity and nose) to areas of “social significance” with a rather wide surface coverage such as the cheeks and the forehead. This phenomenon is possibly supported by selective vasodilation of specific facial sites such as the cheeks leading to greater blood volume retention whereas other regions may vasoconstrict.

### Limitation and Future Directions

In the current study we have employed a novel experimental paradigm for the study of blushing. Blushing as a result of public speaking or being scrutinized in public may have a different physiological intensity and facial thermal values. Nevertheless the overall tendency should be the same. Future studies examining shyness should vary states of shyness in order to be able to differentiate physiologically between the different markers, as they will be able to separate positive shyness such as blushing and negative shyness such as social anxiety and embarrassment. Moreover in the future it would be wise to also examine a male population sample and control for sexual orientation something that was not performed in the current study. In addition it would be interesting to explore using an eye tracker the response that an observer has to a blush in order to further expand our understanding on the facial areas of social significance. What could possibly resolve most of the questions that we have stumbled across in the current study would be to control for individuals who score high in blushing propensity. Then a stronger and clearer pattern will arise for the definition of the results. In extent the selected method of data extraction is optimal for the analyses of moving individuals and subjects since it accounts for movement artifacts. Nevertheless this method not only is laborious but also not ideal for the current experiment. The individuals were rather static throughout the procedure and it would have been ideal to implement tracking algorithms for the current experimental paradigm^2^. Tracking can increase temporal resolution since manual extraction may miss small transient arousal changes. Thus to compensate for micro-alterations in complex physiological phenomena (such as blushing) a higher spatial resolution infrared sensor and a good tracker would be ideal (Zhu et al., 2007). One important limitation of the current study was the small sample size. Although the general tendency of the temperature at the group level was quite robust results should be approached with caution. All data used for the current study will be made publicly available through Dryad Digital Repository^3^ so that scientist can make their own interpretation on the research findings. Moreover since it is the first time that light spectrum analyses was used in emotional research it would be beneficial to verify this technique using a spectrophotometer (Whitehead et al., 2012). Individuals with developmental or mental problems could illustrate different physiological markers after receiving a compliment.

## Conclusion

Overall paying a compliment to an individual raised the temperature of the face. This rise was localized on the cheeks, chin, and forehead whereas other regions such as the periorbital region showed the opposite effect. Blushing of the cheeks and the temperature decrease on the peri-orbital region and the nose may indicate a possible micro-physiological mechanism redirecting blood from the naso-ocular cavity to the cheeks. The visual display of a blush was relatively instant whereas temperature build up on the skin had a delayed response. Blushing may occur as a physiological reflex; nevertheless it does not always reach awareness instantly. This delay is the result of a delay in the heat conduction of the skin. Conversely subjective physiological measures should be interpreted with caution, as they are not the result of a biological but rather a social feedback imprinted in a physio-social response mechanism. Heat is an undeniable element of blushing, however, self-beliefs seem to be the driving forces behind the exhibition of a blush rather than conscious perception of visceral responses.

## Author Contributions

Conception of the experimental protocol: SI, PM, and VR. Writing and editing: SI and PM. Data analyses: SI, PM, and MB. Graphic Illustrations: SI. Concepts and hypotheses: SI and VG.

## Conflict of Interest Statement

The authors declare that the research was conducted in the absence of any commercial or financial relationships that could be construed as a potential conflict of interest.

## Footnotes

1 http://www.guide-infrared.com

2 http://cpl.uh.edu/software/otacs/

3 http://datadryad.org/
